# Supplementary material for: Multifunctional Hybrid MoS2-PEGylated/Au Nanostructures with Potential Theranostic Applications in Biomedicine
Source: Nanomaterials (Basel). 2022 Jun 15;12(12):2053. doi: 10.3390/nano12122053 (PMC9227389; doi:10.3390/nano12122053)
Supplement: Supplementary file 1 [file nanomaterials-12-02053-s001.zip › nanomaterials-1751691-supplementary.pdf]

*nanomaterials*

## Supporting Information

# Multifunctional Hybrid $MoS_2$-PEGylated/Au Nanostructures with Potential Theranostic Applications in Biomedicine

**Thiago R. S. Malagrino [1], Anna P. Godoy [1], Juliano M. Barbosa [1], Abner G. T. Lima [1], Nei C. O. Sousa [1], Jairo J. Pedrotti [1], Pamela S. Garcia [1], Roberto M. Paniago [2], Lídia M. Andrade [2], Sergio H. Domingues [1,3], Wellington M. Silva [4] and Hélio Ribeiro [1] and Jaime Taha-Tijerina [5,6,*]**

[1] Engineering School, Mackenzie Presbyterian University, Rua da Consolação 896, São Paulo 01302-907, SP, Brazil; thiago.malagrino@hotmail.com (T.R.S.M.); annapsgodoy@hotmail.com (A.P.G.); juliano.barbosa@mackenzie.br (J.M.B.); abn.guilherme@gmail.com (A.G.T.L.); nei.sousa@mackenzie.br (N.C.O.S.); jairojpedrotti@gmail.com (J.J.P.); pamela.s.garcia@gmail.com (P.S.G.); sergio.domingues@mackenzie.br (S.H.D.); helio.ribeiro1@mackenzie.br (H.R.)

[2] Departamento de Física, Universidade Federal de Minas Gerais, Avenida Presidente Antônio Carlos, 6.627, Belo Horizonte MG 31270-901, Brazil; paniago@fisica.ufmg.br (R.M.P.); lidia.nanobmrg@gmail.com (L.M.A.)

[3] MackGraphe, Mackenzie Institute for Advanced Research in Graphene and Nanotechnologies, Rua da Consolação 896, São Paulo SP 01302-907, Brazil

[4] Departamento de Química, Universidade Federal de Minas Gerais, Avenida Presidente Antônio Carlos, 6.627, Belo Horizonte 31270-901, MG, Brazil; wellingtonmarcos@yahoo.com.br

[5] Engineering Department, Universidad de Monterrey, Av. Ignacio Morones Prieto 4500 Pte., San Pedro Garza García N.L. 66238, Mexico

[6] Engineering Technology Department, University of Texas Rio Grande Valley, Brownsville, TX 78520, USA

**Citation:** Malagrino , T.R.S.; Godoy, A.P.; Barbosa, J.M.; Lima A.G.T..; Sousa, N.C.O.; Pedrotti, J.J.; Garcia, P.S.; Paniago, R.M.; Andrade, L.M.; Domingues, S.H.; et al. Multifunctional Hybrid $MoS_2$-PEGylated/Au nanostructures with Potential Theranostic Applications in Biomedicine. *Nanomaterials* **2022**, *12*, 2053. https://doi.org/10.3390/nano12122053

Academic Editor: Run Zhang

Received: 17 May 2022
Accepted: 9 June 2022
Published: 15 June 2022

**Publisher's Note:** MDPI stays neutral with regard to jurisdictional claims in published maps and institutional affiliations.

**Copyright:** © 2022 by the authors. Submitted for possible open access publication under the terms and conditions of the Creative Commons Attribution (CC BY) license (https://creativecommons.org/licenses/by/4.0/).

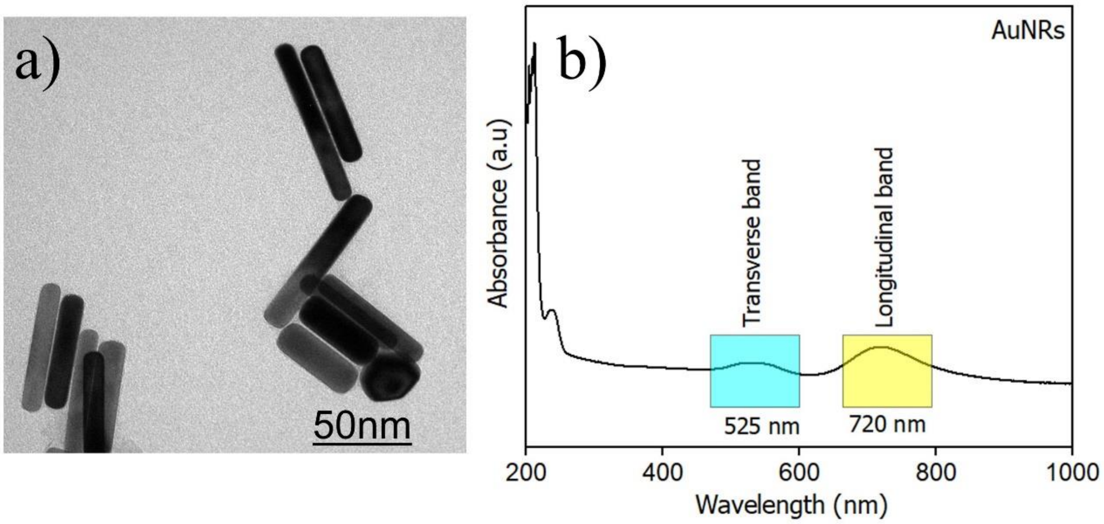


**Figure S1.** (**a**) TEM images of Au nanoparticles (AuNPs) and (**b**) UV-Vis of Au nanorods (AuNRs) suspension.

Figure S1a shows gold nanoparticles TEM image, where the major fraction of gold nanorods AuNRs appears with an average length size ~ 50 nm. However, results also confirmed that small fraction of spherical nanoparticles, as noted by Steckiewicz at al [62]. The gold nanoparticles also were characterized by UV–Vis spectroscopy in range of 200–1000 nm (Figure S1b). The AuNPs showed a characteristic optical feature usually related to as localized surface plasmon resonance (LSP). For the produced AuNRs, typically two plasmon resonances are observed. The transverse and longitudinal LSPR extinction peaks located around 525 and ~720 nm, respectively [63,64]. However, the absorption band at ~530 nm may also be related to the presence of spherical Au nanoparticles [62], which is also confirmed by TEM image. In this case, the position and intensity of the LSPR band depends strongly on the size and surface morphology of gold nanoparticles. The presence of a transverse and longitudinal plasmon resonances is strong evidence that the formation of AuNPs occurred. The aspect ratio of gold nanorods is about 22.8 (length ~80 nm and width ~3.5 nm).

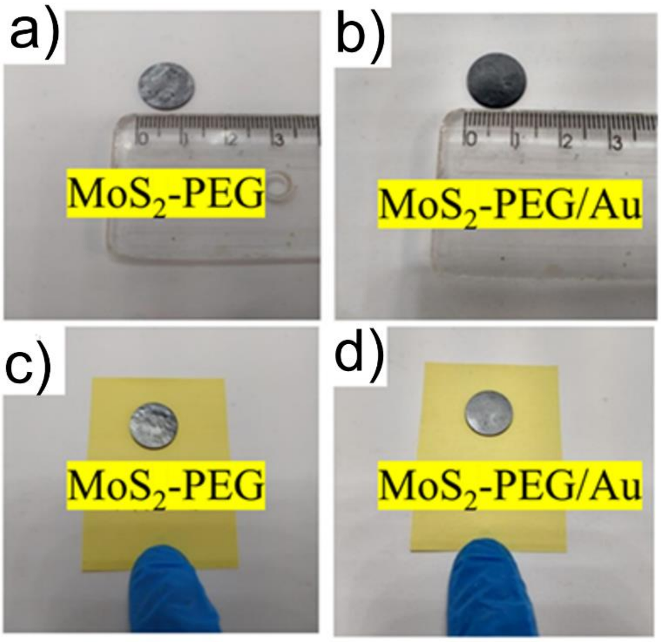


**Figure S2.** Image tablets or electrodes $MoS_2$-PEG (**a**,**c**) and $MoS_2$-PEG/Au (**b**,**d**).

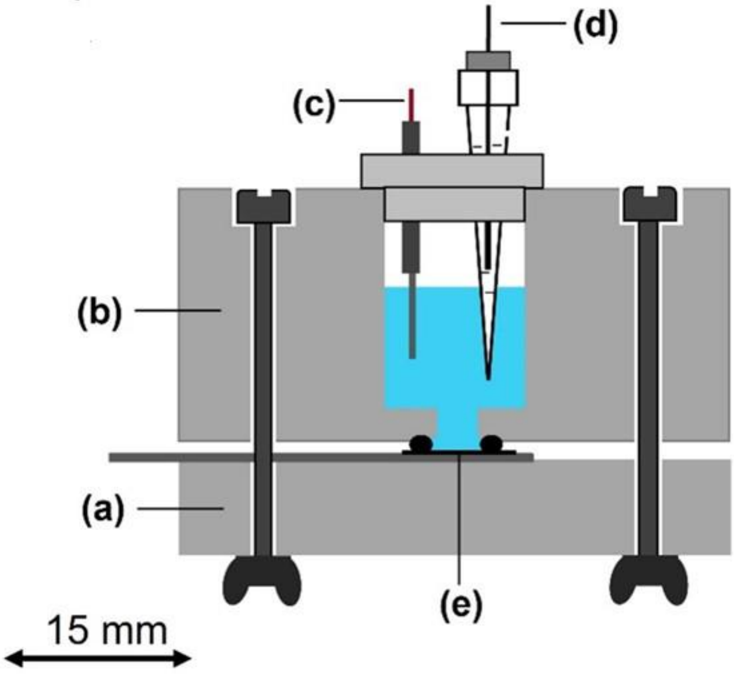


**Figure S3.** Electrochemical cell design: (**a**,**b**) plexiglass pieces, (**c**) auxiliary electrode, (**d**) Ag/AgCl reference electrode and (**e**) working electrode.

The sheet resistance and electrical conductivity of the hybrid nanomaterials method was intended for thin films, a correction factor must be accounted for whenever the sample thickness exceeds 40% of the space between probes. This being the case, for a circular sample measured at its center, the correction factor was calculated by the following equation:

$$C_{circular} = \frac{\ln(2)}{\ln(2) + \ln\left(d^2/_{s^2} + 3\right) - \ln\left(d^2/_{s^2} - 3\right)} \quad (1)$$

where $d$ is the sample diameter and $s$ is the distance between probes. The conductivity was determined by the sheet resistance and the thickness of the samples.

The cell consists of two pieces of plexiglass (a and b) that make up the top and bottom of the device. On the top of 48 × 40 × 40 mm block (b) was made a central orifice with two inner diameters extending throughout the acrylic block to allocate the electrolyte solution. A silicone cap was used on the top of the central hole to set up the lab-made miniaturized Ag/AgCl reference electrode and the auxiliary electrode of platinum in the electrochemical cell with 4 mL internal volume [65]. The working electrode was positioned under pressure between the plexiglass blocks by four 3.2 mm thick and 5.0 mm long brass round head slotted screws. A Viton O-ring with an inner diameter of 2 mm was inserted in the base of the plexiglass piece (b) to limit the geometrical area of the working electrodes and prevent leakage of the electrolyte. The specific capacitance of the electrodes was evaluated in $Na_2SO_4$ 1. 0 mol $L^{-1}$ from cyclic voltammetry (CV) measurements according to the following equation:

$$C_{sp} = \frac{\int i.dv}{2.m.\Delta V.v} \quad (2)$$

where $C_{sp}$ (F $g^{-1}$) is the specific capacitance, $\int i.dv$ is the integrated area of CV curves, $m$ (g) is the active mass of electrode material, $\Delta V(V)$ is the potential window and $v$ (V/s) is the scan rate. The electrochemical measurements were made at 22 ± 0.2 °C room temperature. The voltammograms were measured in triplicate and the last CV was used for capacitance calculations. The contact angle measurements were carried out in a room with ambient controlled conditions (temperature 22 ± 0.2 °C and humidity under 60%) by using a *Drop Shape Analysis System DSA100* modular type from *A.Krüss Optronic GmbH* (Hamburg, Germany) provided with a sample holder horizontally aligned and the *Calibre* software controlled the image processing. After setting the sample on the holder, a micropipette loaded with deionized water without any air bubbles was used to deposit a drop of 10 μL, and take 20 successive images of the advancing contact angle (ACA). Afterward, half of the ultra-pure water droplet volume was removed, and another 20 measurements of the receding contact angle (RCA) were taken. Each material had this procedure repeated three times at three different points of each sample. The estimated contact angle value was the arithmetic mean of the values found on all measurements.

VeroCCL-81 cells were exposed to different concentrations of $MoS_2$-PEG and $MoS_2$-PEG/Au respectively for 24 h and 48 h. The panel below shows representative transmitted images of these cells and nanomaterials agglomeration scattered in some fields. The morphology of cells was not altered by the presence of $MoS_2$-PEG or $MoS_2$-PEG/Au compared to control cells.

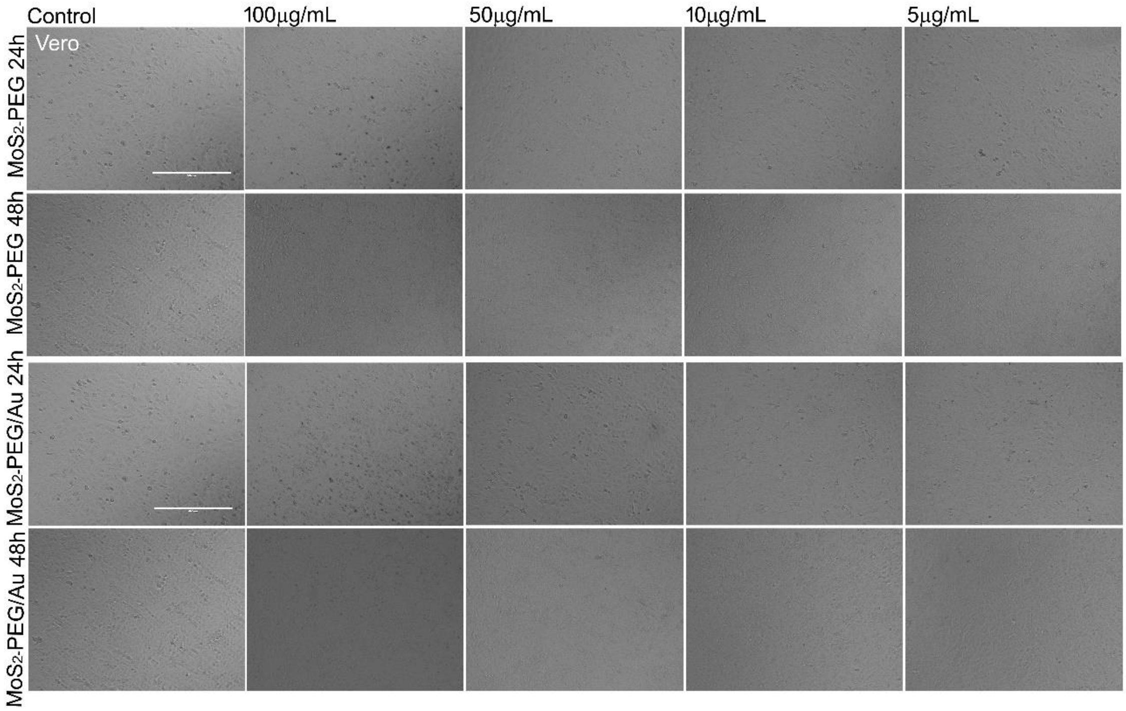


**Figure S4.** Vero cellular morphology. Representative Images show Vero cells exposed to four concentrations of these nanomaterials at 24 h and 48 h. (Bar scale = 400 µm, transmitted image in 10× objective).

A431 squamous cell carcinoma cell lines were used as tumoral model to evaluate $MoS_2$-PEG and $MoS_2$-PEG/Au cellular viability, respectively, for 24 h and 48 h. The panel below shows representative transmitted images of these cells and nanomaterials agglomeration scattered in some fields. The morphology of cells was not altered by the presence of $MoS_2$-PEG or $MoS_2$-PEG/Au compared to control cells.

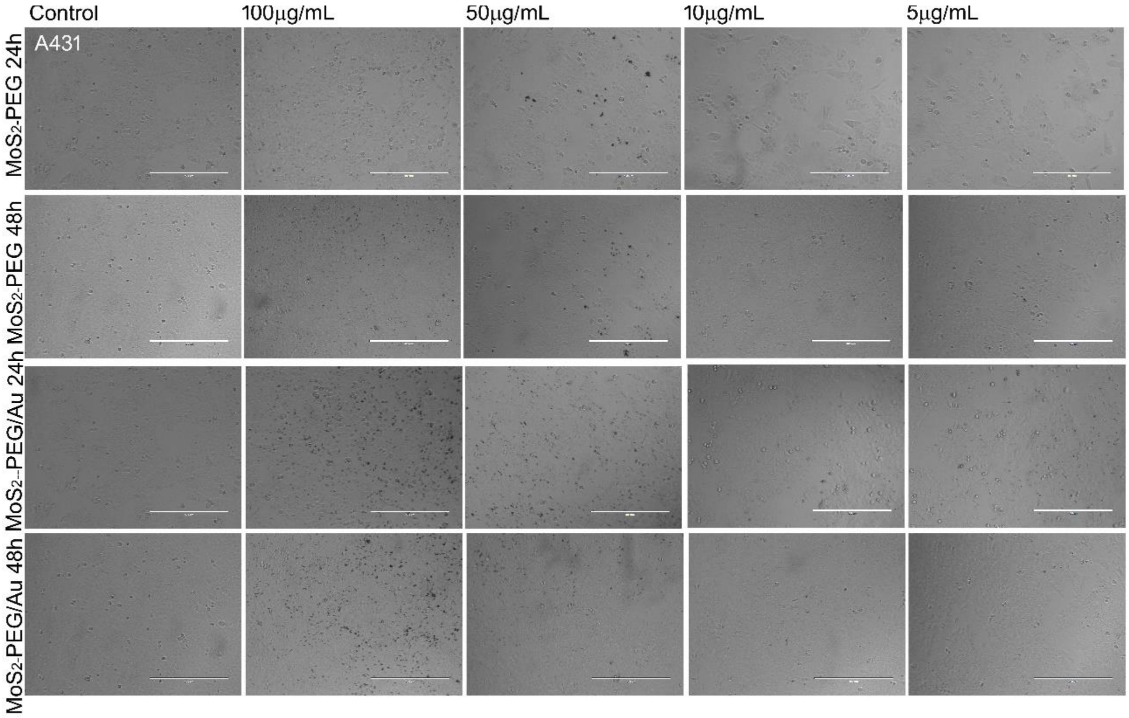

**Figure S5.** A431 cellular morphology. Representative Images show A431 cells exposed to four concentrations of these nanomaterials at 24 h and 48 h. (Bar scale = 400 μm, transmitted image in 10× objective).

Human pharynx carcinoma FaDU cell line was used as a second tumoral model of $MoS_2$-PEG and $MoS_2$-PEG/Au cytotoxicity effects for 24 h and 48 h, respectively. The panel below shows representative transmitted images of these cells and nanomaterials agglomeration scattered in some fields. The morphology of cells suggests decreasing number of cells and differences in their shapes by the presence of $MoS_2$-PEG or $MoS_2$-PEG/Au when compared to control cells.

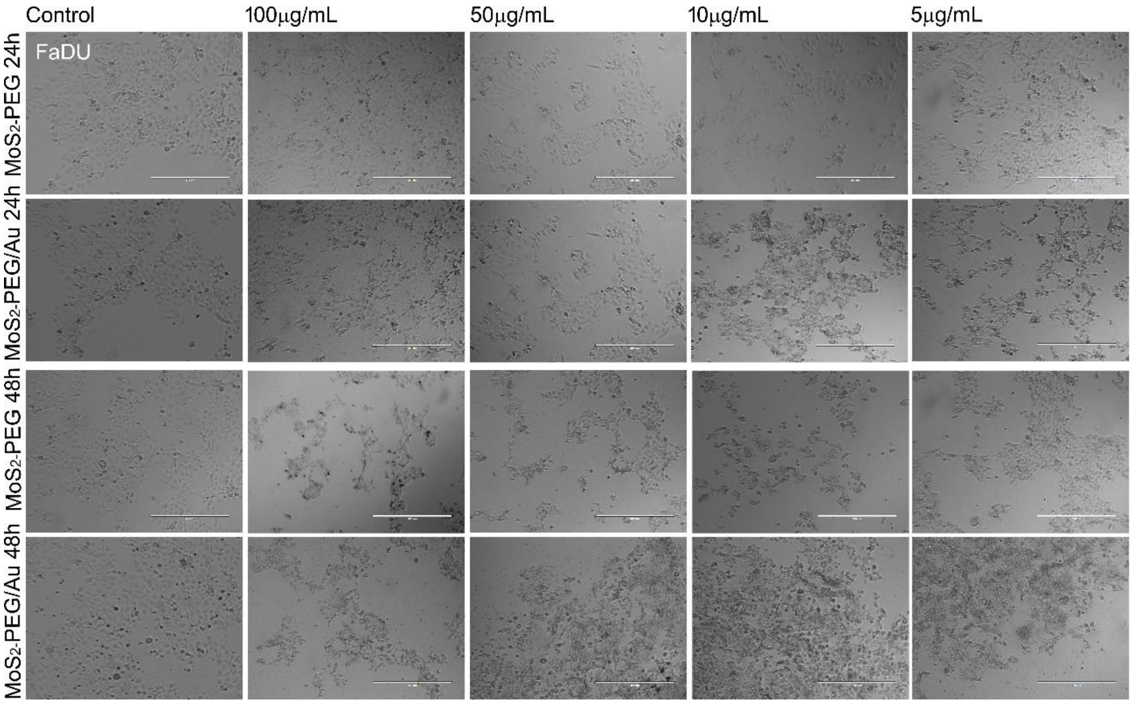


**Figure S6.** FaDU cellular morphology. Representative Images show FaDU cells exposed to four concentrations of these nanomaterials at 24 h and 48 h. (Bar scale = 400 μm, transmitted image in 10× objective).

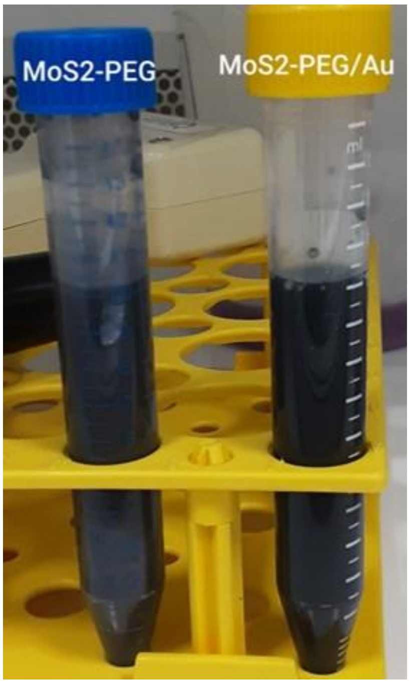


**Figure S7.** $MoS_2$-PEG and $MoS_2$-PEG/Au suspensions (1.0 mg/mL).

## References


62. Steckiewicz, K.P.; Barcinska, E.; Malankowska, A.; Zauszkiewicz–Pawlak, A.; Nowaczyk, G.; Zaleska-Medynska, A.; Inkielewicz-Stepniak, I. Impact of Gold Nanoparticles Shape on Their Cytotoxicity against Human Osteoblast and Osteosarcoma in in Vitro Model. Evaluation of the Safety of Use and Anti-Cancer Potential. *J. Mater. Sci. Mater. Med.* **2019**, *30*, 22. https://doi.org/10.1007/s10856-019-6221-2.
63. Kooij, E.S.; Ahmed, W.; Hellenthal, C.; Zandvliet, H.J.W.; Poelsema, B. From Nanorods to Nanostars: Tuning the Optical Properties of Gold Nanoparticles. *Colloids Surfaces A Physicochem. Eng. Asp.* **2012**, *413*, 231–238. https://doi.org/10.1016/j.colsurfa.2012.01.041.
64. Zhong, X.; Chai, Y.-Q.; Yuan, R. A Novel Strategy for Synthesis of Hollow Gold Nanosphere and Its Application in Electrogenerated Chemiluminescence Glucose Biosensor. *Talanta* **2014**, *128*, 9–14. https://doi.org/10.1016/j.talanta.2014.03.071.
65. Pedrotti, J.J.; Angnes, L.; Gutz, I.G.R. Miniaturized Reference Electrodes with Microporous Polymer Junctions. *Electroanalysis* **1996**, *8*, 673–675. https://doi.org/10.1002/elan.1140080713.
